# A Performance Comparison of 3D Survey Instruments for Their Application in the Cultural Heritage Field

**DOI:** 10.3390/s24123876

**Published:** 2024-06-15

**Authors:** Irene Lunghi, Emma Vannini, Alice Dal Fovo, Valentina Di Sarno, Alessandra Rocco, Raffaella Fontana

**Affiliations:** 1 National Research Council-National Institute of Optics (CNR-INO), Largo E. Fermi 6, 50125 Firenze, Italy; irene.lunghi@ino.cnr.it (I.L.); emma.vannini@ino.cnr.it (E.V.); alice.dalfovo@ino.cnr.it (A.D.F.); 2 National Research Council-National Institute of Optics (CNR-INO), Via Campi Flegrei 34, 80078 Pozzuoli (NA), Italy; valentina.disarno@ino.cnr.it (V.D.S.); alessandra.rocco@ino.cnr.it (A.R.)

**Keywords:** micro-profilometry, structured-light, phase-shift technique, 3D point cloud processing, depth resolution, profiling

## Abstract

Thanks to the recent development of innovative instruments and software with high accuracy and resolution, 3D modelling provides useful insights in several sectors (from industrial metrology to cultural heritage). Moreover, the 3D reconstruction of objects of artistic interest is becoming mandatory, not only because of the risks to which works of art are increasingly exposed (e.g., wars and climatic disasters) but also because of the leading role that the virtual fruition of art is taking. In this work, we compared the performance of four 3D instruments based on different working principles and techniques (laser micro-profilometry, structured-light topography and the phase-shifting method) by measuring four samples of different sizes, dimensions and surface characteristics. We aimed to assess the capabilities and limitations of these instruments to verify their accuracy and the technical specifications given in the suppliers’ data sheets. To this end, we calculated the point densities and extracted several profiles from the models to evaluate both their lateral (XY) and axial (Z) resolution. A comparison between the nominal resolution values and those calculated on samples representative of cultural artefacts was used to predict the performance of the instruments in real case studies. Overall, the purpose of this comparison is to provide a quantitative assessment of the performance of the instruments that allows for their correct application to works of art according to their specific characteristics.

## 1. Introduction

Three-dimensional modelling and 3D reconstruction methods are becoming increasingly popular in several fields, ranging from industrial metrology [1] (reverse engineering, quality control, micro-measurements) to topographical survey [2] (infrastructure and landscape models), from medicine [3] (surgical planning, diagnoses) to forensics [4] (crime scene reproduction, criminal investigations) and from urban planning [5] (estimation of shaded areas and solar irradiation, surveillance camera positioning) to cultural heritage [6] (digital conservation, remote access, virtual accessibility).

The high accuracy and resolution achieved by the instruments, software and methodologies developed in recent years have made it possible for 3D surveying and modelling to faithfully reproduce objects’ shapes.

Nowadays, there are many devices either available on the market or that have been developed by research groups, the characteristics of which cover a wide range in terms of both resolution (from a few nanometres to metres) and depth of field (from microns to a few kilometres) [7]. The high performance of these instruments has enabled the technology to become more and more widespread for the study of artworks.

As an example, 3D shape and surface reconstruction can provide useful information at both the macroscopic and microscopic levels: it can be used to carry out non-destructive analyses, access objects that are difficult to reach, formulate integration hypotheses through virtual simulations and create high-accuracy copies [8,9]. Moreover, the digitalisation of objects of artistic interest is noteworthy for cultural heritage risk assessment and control (e.g., disasters caused by earthquakes, climate changes, wars and human negligence).

The generation of 3D models is achieved using non-contact systems based either on active or passive techniques, depending on the use of a light source (a laser or a projector) or ambient light, respectively [10]. Furthermore, the generation of a 3D model can be performed through image-based (e.g., photogrammetry) or range-based (e.g., laser scanning) techniques or even a combination of them [11]. The proper choice of technique depends on various factors such as the size of the object, instrument portability, the resolution and accuracy required and time and cost availability. Presently, there are plenty of 3D techniques that are well suited to diagnostics in works of art. Their applicability to different case studies depends on their performance in terms of resolution, gauge volume/area, acquisition time and the instrumentation cost. Given that the restoration of a work of art can last from a few months to several years, it is often necessary to repeat measurements with different instruments at different times in variable contexts (e.g., to document the restoration intervention, a shape survey is carried out before starting the work and after the work is finished) [12]. Moreover, the investigation of artworks usually requires both overall and detailed analyses of the surface, which then requires a multi-modal and multi-resolution approach to assess all issues. Therefore, understanding the quality of the 3D results is essential to know what information can be extracted from the 3D models obtained from different instruments to address the proper choice and consequently the appropriate settings for each device.

Among the active techniques, phase-based scanning, structured-light topography and micro-profilometry provide 3D models from the large to small scales in a non-invasive and non-contact way, which are prerequisites for their application to the field of cultural heritage.

Phase-based scanning uses phase-shift technology: a constant laser beam (infrared light of various wavelengths) is projected outward from the scanner, and upon contact with an object, it is reflected back to the scanner. The distance from the scanner to the object is accurately determined by measuring the phase shifts in the waves of the infrared light [13,14]. The technique is widely used in terrestrial surveys for 3D modelling of buildings, archaeological sites, monuments and big statues [15,16,17].

Structured-light topography is an active triangulation technique based on the projection of non-coherent light patterns (either coloured or monochromatic) that are distorted by the geometric shape of the surface. The principle of structured-light topography is the extraction of the 3D surface morphology based on the information derived from the distortion of the projected pattern, as observed using one or two cameras [18,19]. Thanks to its sub-millimetric resolution [20,21,22], the technique is mainly applied to archaeological finds, statues, panels and mural paintings.

Micro-profilometry is an incoherent holographic technique based on the properties of birefringent crystals [23]. For each point on an object, the interference pattern between the ordinary and extraordinary rays has a scale parameter that is a function of the distance of the point. The technique achieves a micrometric axial and lateral resolution and therefore is well suited to a variety of surveying applications. These include the detection of paint detachments, the study of the craquelure of the pictorial layer, calculation of a surface’s roughness and the documentation of the conservation status and micro-deformation of a surface [24,25].

In this work, we compared the performance of four portable devices for 3D survey that are part of the Heritage Science Group’s (CNR-INO) portfolio: a micro-profilometer (MP), an in-house prototype; the FARO Focus phase-modulation scanner and the MICRON3D and EinScan Pro 2x structured-light scanners. The FARO Focus, MICRON3D and EinScan scanners were recently acquired to upgrade the MOLAB (Mobile LABoratories) platform of the Italian node of the European Research Infrastructure for Heritage Science (E-RIHS.it, https://www.e-rihs.it (accessed on 27 March 2024)). In order to fulfil the platform requirements and provide a general and technical description of all the instruments that allows their applicability to different case studies to be foreseen, we have evaluated the limitations, potentialities and versatility of the devices. This entailed an assessment of their actual lateral and axial resolution, as well as the accuracy of the 3D products. To this end, we scanned four samples, representative of different heritage objects, using all the instruments.

Since the data sheets of the scanners do not supply the axial resolution and, to the best of our knowledge, there is no standard procedure for its calculation, we report in this work a method for its evaluation.

## 2. Materials and Methods

### 2.1. Samples

**Figure 1:**
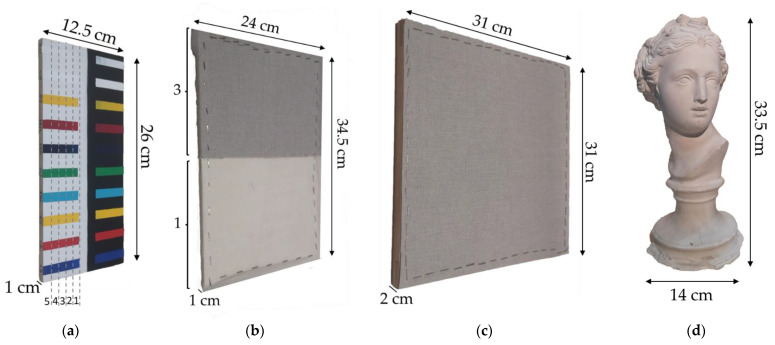
Samples used for 3D reconstruction: (**a**) acrylic paint mock-up: for the ten acrylic colours on the white base, the five areas of increasing thickness are numbered from 1 to 5 and delimited by vertical dotted lines, (**b**) canvases 1 and 3, (**c**) canvas 2 and (**d**) plaster statue.

From the vast panorama of works of art, we selected materials that were representative of the objects that we most frequently analyse. Therefore, several samples made of acrylic pigments spread at controlled and known thicknesses, some canvases of different densities and a plaster statue were chosen to cover different types of artworks of varying colour and form (Figure 1).

The acrylic paint mock-up consists of a wooden support (12.5 cm × 26 cm × 1 cm, Figure 1a), covered half in white and half in black base paint. Ten acrylic paints were applied to each half, and each paint was layered over five 1 cm^2^ adjacent areas by increasing the thickness (from 1 to 5) by about 50 µm each time. A complete characterisation of the latter can be found in ref. [26]. The canvas samples are labelled from 1 to 3 for increasing thickness: canvases 1 and 3 (thinnest and thickest) are mounted onto a wooden frame whose dimensions are 24 cm × 34.5 cm × 1 cm (Figure 1b), with canvas 2 (intermediate thickness) mounted onto a wooden frame 31 cm × 31 cm × 2 cm in size (Figure 1c). The plaster statue (with a base diameter of 14 cm and a height of 33.5 cm, Figure 1d) is a half-bust of a female figure.

### 2.2. Structured-Light Topography: EinScanPro 2x

The EinScan Pro 2x scanner (SHINING 3D Tech. Co., Ltd., Hangzhou, China, https://www.shining3d.com (accessed on 10 January 2024)) [27,28] is a 3D digitising device that generates high-quality 3D models of small and medium-sized objects. Based on the projection of white LEDs, it is equipped with a central projector and two side monochrome cameras located at the extremities of the instrument. An additional 1.3 MP external camera is added for texture acquisition. The acquisition and data processing are controlled by the ExScan Pro 3.7.0.3 proprietary software. Three scanning modes are available: handheld (HD), rapid handheld and fixed scan with or without the aid of an automated turntable. The latter is recommended for high accuracy and high resolution. The working distance is 510 mm, with a depth of field of about 200 mm. In the fixed scan mode set for this study, the single shot accuracy is 0.04 mm, the resolution point distance is 0.16 mm and the scan efficiency is better than 0.5 s [29] (the technical specifications are summarised in Table 1).

### 2.3. Structured-Light Topography: MICRON3D Color

MICRON3D color (SMARTTECH 3D, Warsaw, Poland, www.smarttech3d.com (accessed on 10 January 2024)) [30] is a mobile 3D scanner for the precise digitalisation of colourful objects.

The scanner uses a white LED for structured-light fringe projection and two high-resolution CCD cameras (18 Mpx). Its working distance is 70 cm, and its field of view is 30 cm × 40 cm, with a depth of field of about 20 cm. The distance between points is 80 µm, the density is 150 pp/mm^2^ and the accuracy is 60 µm [31] (the technical specifications are summarised in Table 1). The set includes a stable tripod with a pan/tilt head, positioning lasers, a controlled rotary table for measurement automation, a professional shadeless lighting system and transport cases. A mobile working station delivered with the scanner is equipped with special SMARTTECH3Dmeasure proprietary software v.23 which enables both control over the scanner and user-friendly work with scanned data. The software seamlessly deals with millions of colored scans points: it allows for the simultaneous measurement of the object’s shape and texture, providing a point cloud with both XYZ spatial coordinates and RGB colour values.

The instrument has two working modalities, Precise Mode, which uses every pixel of the detector to achieve the highest possible resolution (full resolution), and Simplified Mode, which uses every fourth pixel of the detector, resulting in 25% of the full resolution.

In this work, we chose Precise Mode to recreate the finest details with great accuracy to assess the best performance of the instrument. To avoid shaded areas, we used two professional shadeless lighting systems equipped with softboxes, placed at 45° from the object’s surface.

### 2.4. Phase-Modulation Technique

FARO Focus Premium (FARO is headquartered in Lake Mary, FL, USA, www.faro.com (accessed on 10 January 2024)) [32,33] is a portable phase-modulation scanner for the digitalisation of large-scale objects, e.g., buildings and archaeological sites, suited for both indoor and outdoor measurements. It uses a class 1 laser at 1553.5 nm and an HDR camera (266 Mpx colour resolution) coaxial with the scanning mirror, allowing for a 300° vertical/360° horizontal field of view (FOV), with 0.009° sampling steps in both directions. The depth of field ranges from 0.5 m to 350 m. The distance between points is 1.5 mm within a scanning distance of 10 metres, with a 2 mm accuracy [34] (the technical specifications are summarised in Table 1). The instrument is supplied with the SCENE proprietary software v.2022.1.0 for data acquisition and processing.

In this work, we set the sample-to-instrument distance at 70 cm and the resolution and quality parameters at their maximum values, i.e., 1/1 and 5×, respectively. We narrowed the field of view to capture only the scanning object, and the resulting acquisition time was about ten minutes.

### 2.5. Laser-Scanning Micro-Profilometry

The optical micro-profilometer (MP) is an in-house laser scanning device developed at INO-CNR [35,36] made of a commercial probe (ConoPoint-10 by Optimet, Jerusalem, Israel) mounted onto two motorised high-resolution (precision of 0.1 μm) linear stages, perpendicularly assembled. The maximum scanning area is 300 × 300 mm^2^, and the scanning speed ranges from 100 to 400 points/s depending on the sampling step and the maximum travel length; the system is computer-controlled.

The probe is equipped with a set of lenses with different focal lengths, resulting in different working distances, spot sizes and resolutions. In this work, we used 50, 100 and 200 mm focal lengths (hereinafter referred to as f50, f100 and f200, respectively), whose technical specifications are summarised in Table 1.

The acrylic mock-ups and the canvas samples were acquired with all the objectives. In contrast, the 50 mm lens was not employed for the plaster statue (Figure 1d) due to its short working distance and field depth, which prevented the acquisition of the surface morphology. We chose sampling steps of 20, 50, 100 and 200 µm (hereafter referred to as s20, s50, s100 and s200, respectively). To avoid an excessive scanning time, we acquired selected ROIs (region of interest) on the samples, whose dimensions are summarised in Table 2 with the corresponding acquisition parameters.

For all scanners, we performed a single acquisition on each sample, without filling the holes. The point clouds were cleaned to remove noise and undesired points and then transformed into a triangle mesh. All the point clouds and meshes were exported without texture.

## 3. Results

### 3.1. Three-Dimensional Data Quality Comparison

For each sample and each instrument, the details of the measured areas and the related point clouds are summarised in Table 3. Concerning the micro-profilometer, we report one example for each sampling step (20, 50, 100, 200 µm) since the point density is set by the user and is independent of the focal length.

**Figure 2:**
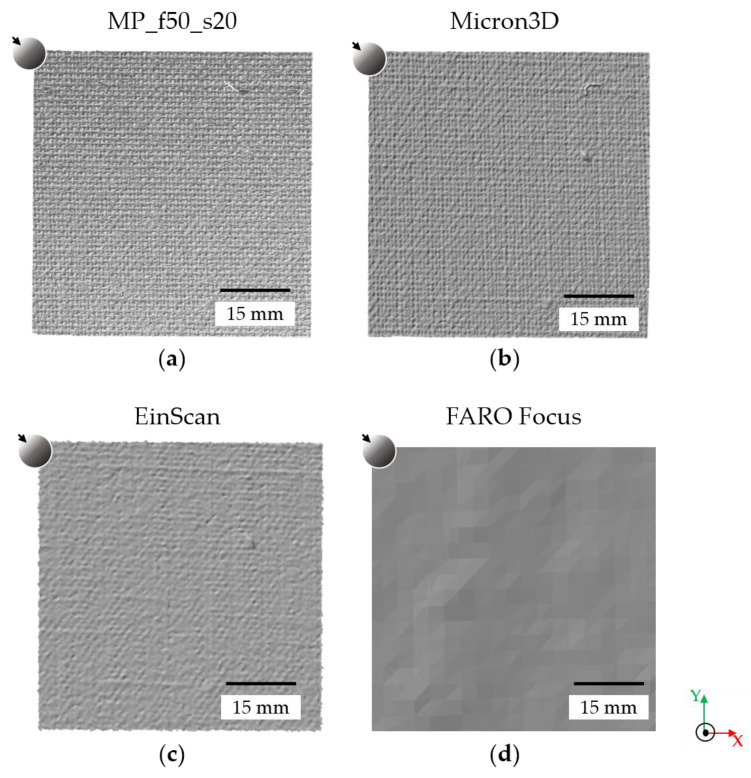
Sample canvas 2: 3D outcomes generated by (**a**) MP with 50 mm focal length and 20 µm sampling step; (**b**) MICRON3D scanner; (**c**) EinScan scanner; (**d**) FARO Focus scanner.

For a qualitative visualisation of the different performances of the four instruments in three-dimensional rendering, Figure 2 shows the 3D outcomes obtained on sample canvas 2. To assess the quality of the models and to compare the results obtained with the different instruments, we computed the point density (pp/mm^2^) of the clouds [37]. The point density is defined as the number of points per unit area, and it is directly related to the point spacing (the closer the points are, the higher the point density). To calculate the point density, we used a specific tool in the CloudCompare software (v.2.12.4, https://www.danielgm.net/cc/ (accessed on 15 February 2024)) based on the following formula:

(1) $$Density=\frac{N}{\pi R^{2}}\;,$$

where *N* is the number of neighbours for each point within a sphere of radius *R*.

**Figure 3:**
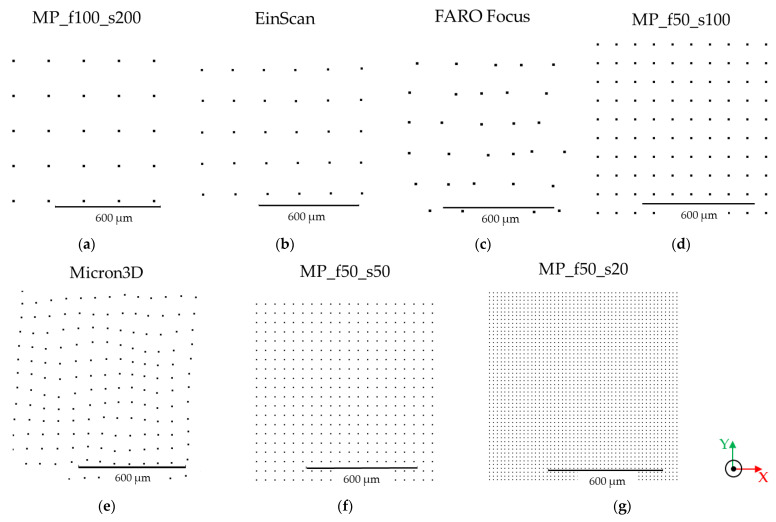
Point cloud details of sample canvas 2 generated by (**a**) 100 mm focal length and 200 µm sampling step (24 ± 2 pp/mm^2^); (**b**) EinScan scanner (27 ± 1 pp/mm^2^); (**c**) FARO Focus scanner (30 ± 3 pp/mm^2^); (**d**) 50 mm focal length and 100 µm sampling step (95 ± 7 pp/mm^2^); (**e**) MICRON3D scanner (145 ± 10 pp/mm^2^); (**f**) 50 mm focal length and 50 µm sampling step (375 ± 25 pp/mm^2^); (**g**) 50 mm focal length and 20 µm sampling step (2448 ± 178 pp/mm^2^).

The software tool gives the density mean value and the corresponding standard deviation, which are reported in the caption of Figure 3, showing zoomed areas of the point clouds from sample canvas 2 to graphically visualise the point density generated by each instrument.

The point density of the 3D models generated with micro-profilometry is independent of the surface morphology and is known a priori once the sampling step is set (Figure 3a,d,f,g). On the contrary, models generated with the scanners have different density values depending on the sample; therefore, the point density is determined by the surface morphology and/or the colour of the sample. 

Among the scanners, MICRON3D exhibits the best performance: the measured point density ranges from 125 ± 11 to 145 ± 10 pp/mm^2^ on sample canvases 1 and 3 and sample canvas 2, respectively, which is slightly smaller than the 150 pp/mm^2^ value provided by the manufacturer. The point density generated with EinScan ranges from 26 ± 2 pp/mm^2^ on sample canvases 1 and 3 to 27 ± 6 pp/mm^2^ on the plaster statue, which is lower than the theoretical value computed from the given point spacing of 0.16 mm, following the formula:

(2) $$number\;of\;points=\frac{unit\;area\;\left(mm^{2}\right)}{{\left[distance\;between\;points\;\left(mm\right)\right]}^{2}}\;$$

Therefore, a point density of approximately 39 pp/mm^2^ can be estimated by dividing the number of points by the unit area. Finally, the point density generated with FARO Focus ranges from 25 ± 5 to 35 ± 11 pp/mm^2^ for the acrylic paint mock-up and the plaster statue, respectively, which is greater than the 1 pp/mm^2^ theoretical value given a point spacing of 1.5 mm (following Equations (1) and (2)). This overestimation can be ascribed to the high noise level of the clouds (see Section 3.2 below).

**Figure 4:**
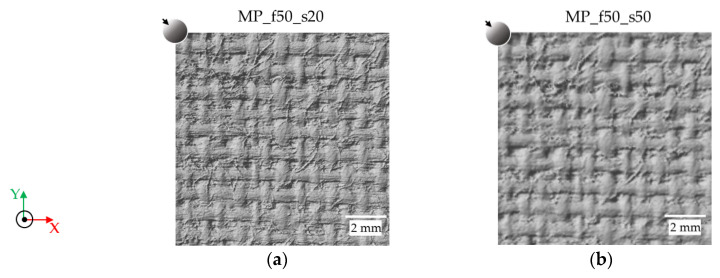

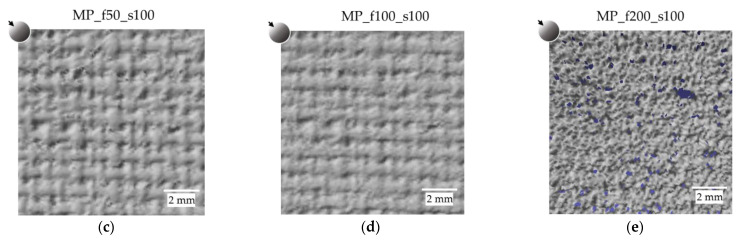
Details of the meshes of sample canvas 2 generated by micro-profilometry with 50 mm focal length and (**a**) 20 µm and (**b**) 50 µm sampling steps and 100 µm sampling steps and (**c**) 50 mm, (**d**) 100 mm and (**e**) 200 mm focal lengths.

Concerning the micro-profilometer, the instrument performance varies with the focal length of the objective lens and the sampling step selected by the user. As an example, Figure 4 shows some meshes of sample canvas 2 generated by varying the latter characteristics. The meshes, shown in greyscale, were generated using the MeshLab software (v.2022.02 https://www.meshlab.net/ (accessed on 15 February 2024)) and choosing the same direction for the impinging light (from the top left) to highlight the features of the surface. Figure 4a,b display the models obtained with the 50 mm lens and sampling steps of 20 µm and 50 µm, respectively. The 50 mm focal length sets a spot size of a nearly 37 µm diameter; therefore, the 20 µm sampling step entails oversampling, which results in a clearer visualisation of the canvas warp and weft. A better lateral resolution is obtained at the expense of a significantly higher acquisition time: for the 60 × 60 mm^2^ area, the scanning time is 90 and 36 min for 20 µm and 50 µm, respectively.

Since the choice of lens focal length determines not only the spot size but also the working distance and axial resolution, we analysed the models acquired with the same sampling step (100 µm) but with three different focal lengths, i.e., 50, 100 and 200 mm (Figure 4c–e, respectively). The 200 mm lens (Figure 4e) is unable to provide a good model, as no surface features (canvas threads) are detected, and several holes are present. On the contrary, the canvas texture is visible in both the 50 and 100 mm lens models, although the former lens allows for better visualisation of the details. When measuring an artwork, the choice of the best-suited lens depends on the surface morphology of the object to be scanned: the depth of focus and the stand-off distance play a major role.

### 3.2. Profiling

The extraction of a cross-sectional profile [38] is a useful criterion for evaluating and comparing the performance of the different instruments: it allows for visualisation of the 3D points on a plane, providing well-detailed geometric features. We performed the profile extraction in MATLAB.

#### 3.2.1. Plaster Statue

**Figure 5:**
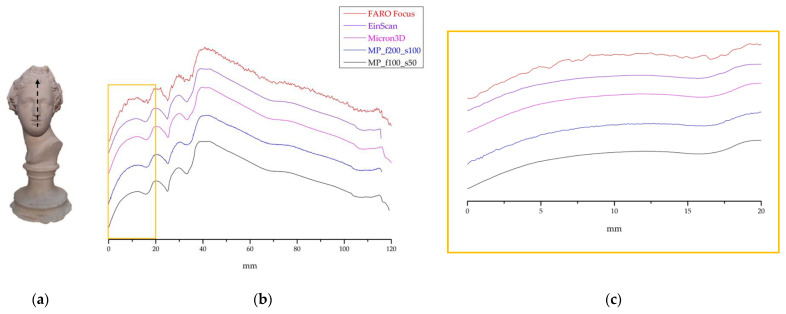
(**a**) Plaster statue, with the dotted arrow indicating the acquisition direction of the profiles shown in (**b**) and in detail in (**c**).

For profile evaluation, we imaged the plaster statue using FARO Focus, EinScan, MICRON3D and the MP equipped with 200 mm and 100 mm lenses. From the point clouds, we extracted a profile along the black dotted line in Figure 5a, following the arrow direction. As shown in Figure 5b, all the instruments can detect the shape of an object with a complex 3D geometry. The cloud collected with the FARO Focus is the noisiest, as the instrument is not designed for surveying small to medium-sized objects; concerning the micro-profilometer, MP_f200_s100 is noisier than MP_f100_s50 (Figure 5c). Therefore, whenever the object’s shape is within the dynamic range of the 100 mm lens (see Table 1), the latter should be preferred.

#### 3.2.2. Canvas Samples

A challenging issue in the cultural heritage field is gathering surface morphology data for nearly flat objects such as panel or canvas paintings. Specifically, the detection and measurement of tiny details, including canvas texture, small cracks, holes, mended tears and linings [39], are useful to conservators for planning a proper restoration intervention. Therefore, we analysed the 3D rendering of the three canvas samples to evaluate the density of the three canvasses: for each instrument, we extracted a profile (10 mm) in an area of the point cloud where the texture’s features were easier to recognise.

**Figure 6:**
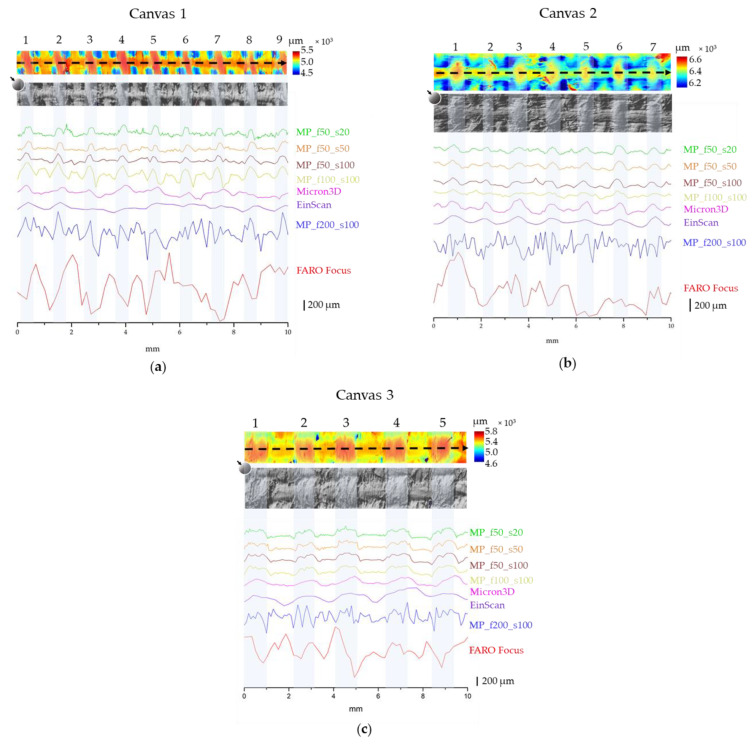
(**a**–**c**) Detail of canvases 1, 2 and 3, respectively. Top: colour map, with the arrow indicating the profile direction, and simulated raking light image for MP_f50_s20; bottom: profiles as measured by the different instruments.

For each canvas, we used the point cloud acquired by MP_f50_s20 as a reference, as the micro-profilometer equipped with the 50 mm lens demonstrated the best resolution and accuracy (see Section 3.1). Figure 6a–c (top) show the details of the colour maps and simulated raking light images of the MP_f50_s20 point cloud for canvases 1, 2 and 3, respectively.

For all the canvases, the micro-profilometer profiles (Figure 6a–c bottom) allow us to recognise the sequence of the warp and weft, except for with the 200 mm lens (MP_f200_s100) cloud, where the noise exceeds the thread thickness. Increasing the sampling step has the effect of smoothing the profile, without affecting the proper reading of the warp and weft heights.

Neither of the structured-light systems (MICRON3D or EinScan) allowed us to identify the warp and weft in canvas 1 (Figure 6a), whereas both scanners detected the texture in canvases 2 and 3 (Figure 6b,c, respectively).

With regard to FARO Focus, due to the poor signal-to-noise ratio of the profiles, none of the canvas shape were recognised.

For each canvas, we calculated the texture density as the number of threads per centimetre using the colour maps, the raking light images and the profiles. The density of canvases 1, 2 and 3 resulted in 9 threads/cm, 7 threads/cm and 5 threads/cm, respectively.

#### 3.2.3. Acrylic Paint Mock-Up

**Figure 7:**
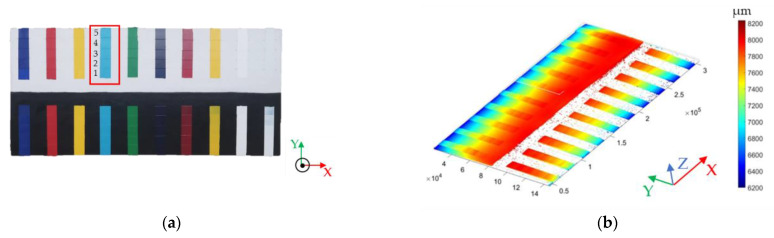

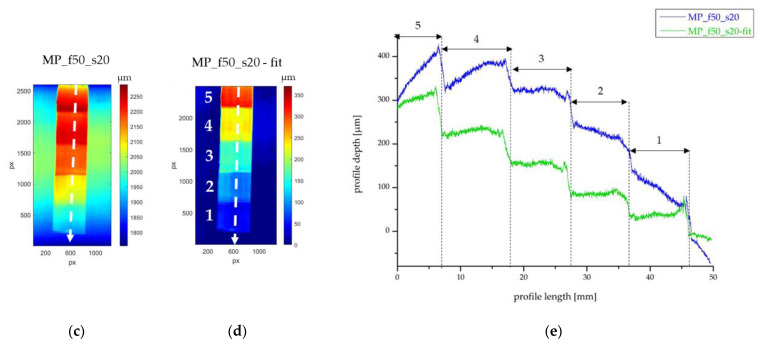
(**a**) Acrylic paint mock-up with the red rectangle highlighting the analysed area (light blue pigment). Colour map of (**b**) the MP_f50_s50 point cloud, (**c**) the selected area on the raw point cloud and (**d**) its conditioned surface. The white dotted arrows in (**c**,**d**) show the direction along which the profiles were extracted, which are plotted in blue and green, respectively (**e**). The five areas of increasing thickness are numbered from 1 to 5 in (**a**,**d**,**e**).

For all the instruments used in this work, no data sheet specifies the axial resolution value. To assess the instrument performance in quantifying this parameter, we extracted a set of profiles from the point clouds acquired on the acrylic paint mock-up, the thickness of which is known [26] (Figure 7a).

None of the instruments gave any results on black pigments, as can be seen in Figure 7b, which shows the colour map of the MP_f50_s50 point cloud, where the lack of points corresponds to the black area. For axial resolution computation, we selected the light blue pigment layered onto the white base (highlighted in red in Figure 7a). The wooden support warp, clearly visible in the colour map (Figure 7b), was removed by subtracting the second-order best-fitting curve from the raw data. The raw and conditioned surfaces from the MP_f50_s20 point cloud are shown in Figure 7c,d, respectively.

The profile extracted along the white arrow (Figure 7c,d) is shown in blue and green for the raw and conditioned surfaces, respectively (Figure 7e). The former is affected by the warp of the support, whereas the latter has the expected step trend.

**Figure 8:**
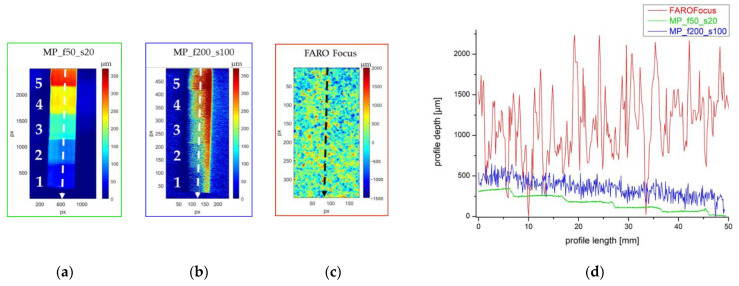

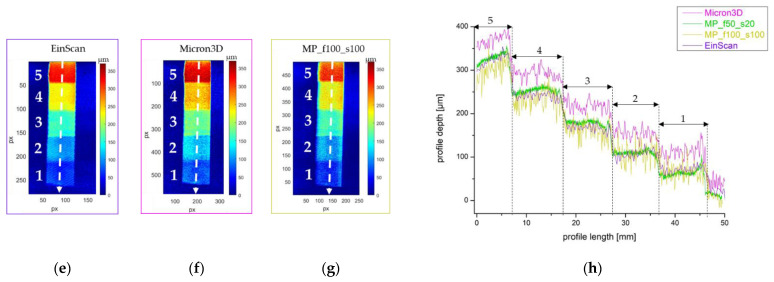
Conditioned surfaces of (**a**) MP_f50_s20, (**b**) MP_f200_s100, (**e**) EinScan, (**f**) MICRON3D and (**g**) MP_f200_s100 point clouds; (**c**) raw colour map of FARO Focus point cloud. The dotted arrows in (**a**), (**b**,**c**) indicate the direction of the extracted profiles plotted in (**d**), while the white dotted arrows in (**a**,**e**–**g**) indicate the direction of the extracted profiles plotted in (**h**).

Since the objective focal length determines the axial resolution (besides the lateral one and the depth of field (DOF); see Table 1), we analysed the MP point cloud for each lens (50 mm, 100 mm and 200 mm focal lengths). Again, we used the MP_f50_s20 point cloud as a reference. As previously observed for the canvas samples, the signal-to-noise ratio for both the FARO Focus and MP_f200_s100 profiles does not allow us to detect any difference in the thickness of the five pigment areas (Figure 8b–d). FARO Focus does not even detect the deformation of the support: the surface appears to be flat (Figure 8c); therefore, no point cloud best fit was necessary.

The EinScan, MICRON3D and MP_f100_s100 profiles show a step trend that allows us to measure all the paint thicknesses, enabling us to state that the instrument axial resolution is better than 50 µm (Figure 8e–h). MICRON3D overestimates the thicknesses of the acrylic layers, as its profiles are higher than the others.

**Figure 9:**
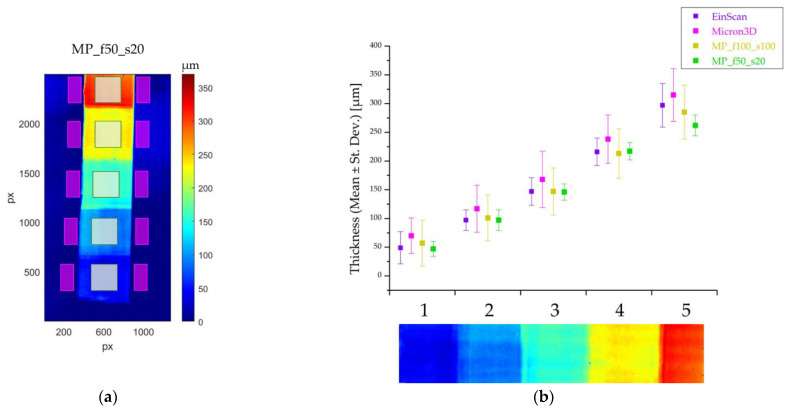
(**a**) Conditioned surface of the MP_f50_20 cloud with the areas selected for the thickness computation highlighted in light green on the pigment and in purple at the sides on the preparation layer. (**b**) Mean thickness and standard deviation for each painted area, shown on the *x*-axis in a colour scale.

We calculated the mean value and the standard deviation of the quota values of a 6 × 6 mm^2^ area on the conditioned surface in the centre of each painted region (from 1 to 5) and in two 6 × 3 mm^2^ areas at the sides of each region on the preparation layer (Figure 9a) to obtain the mean value and the error for each layer thickness. The resulting thickness values with their standard deviation are plotted in Figure 9b and reiterated in Table 4, where the theoretical values are reported for reference. Taking into account the handmade nature of the mock-ups, the values are in accordance with those expected.

The mean values obtained from the MICRON3D clouds confirm that the instrument overestimates the thickness in all the examined regions. The results obtained by MP_f50_s20, MP_f100_s100 and EinScan are quite similar, with the greatest dispersion in region 5. This effect may be due to the uneven thickness of the area, as evidenced by the rising profile graph in Figure 8h.

## 4. Discussion

In this work, we evaluated the performance of a set of 3D instruments, both commercial and developed by the INO’s Cultural Heritage Group: an in-house micro-profilometer, the FARO Focus phase-modulation scanner and the MICRON3D and EinScan Pro 2x structured-light scanners.

To this end, we analysed the quality and geometric accuracy of the point clouds by calculating the point density and analysing some significant profiles. The axial resolution, a parameter that is not given in any of the instruments’ data sheets, was assessed by measuring pigment samples of a known thickness.

The micro-profilometer provides a point cloud on a regular grid whose pitch is given by the sampling step. Therefore, the theoretical point density can be computed a priori, which is consistent with the calculated value. An objective lens of a 50 mm or 100 mm focal length ensures high transversal and axial resolution, which allow for a good reproduction of nearly flat objects with very small details, whereas the lens with a 200 mm focal length has a poor signal-to-noise ratio. On the other hand, the two former lenses have a tiny measurement range, making them ineffective for capturing objects with complex shapes (e.g., big extrusions/intrusions). The acquisition time of the MP is considerably longer than that of the other instruments.

The structured-light instruments (EinScan and MICRON3D) are well suited to the survey of objects with a complex geometry: the two instruments showed great versatility in the detection of both small details, such as the canvas texture, and pictorial layer thickness, having an axial resolution better than 50 µm. The values measured with the MICRON3D scanner were slightly overestimated.

The FARO Focus scanner, despite being designed for surveying large-scale objects such as monuments and buildings, also had a good performance in the survey of small objects such as the plaster statue. It was ineffective for the reproduction of details such as the canvas texture and the pigment thicknesses due to its poor signal-to-noise ratio. Besides the technical specifications, when choosing an instrument for the survey of a cultural heritage object, other issues have to be taken into account, such as the equipment cost, acquisition and processing time and instrument portability and versatility. To date, the cost of 3D instrumentation is quite high and typically increases with increasing device performance. The price range of structured-light scanners is quite wide, and in the present case, the MICRON3D scanner is much more expensive than EinScan; the MP cost stands between the two, and FARO is about the same price as MICRON3D. Even though artworks are stationary, apparently making the acquisition time a worthless consideration, the hourly cost of specialised personnel has to be considered, making the acquisition and processing times important parameters. Except for the micro-profilometer, whose acquisition time ranges from tens of minutes to several hours depending on the measurement parameters (scanning area, sampling step; see Table 2), the other three scanners have a scanning speed in the order of seconds/minutes, depending on the resolution and data quality required. Finally, the ease of transport and use outside a laboratory is an essential requirement in the cultural heritage field since moving artworks is extremely difficult and often impossible, both because of their safety and the onerous costs involved. In terms of versatility for in situ measurements, the micro-profilometer is the least versatile, whereas the other scanners, given their lightness and manageability, are all suitable for measurements even in impervious conditions.

In future works, we will evaluate the quality of the complete 3D digital models produced with the different instruments, trying to assess the quality of the texture rendering.

## 5. Conclusions

In this paper, we presented a comparison of the performance of four instruments for the 3D survey of samples simulating objects of historical and artistic interest. This comparison was mainly based on the scanners’ characteristics that allow for a good reproduction of almost flat objects, such as wood, canvases and mural paintings, which are the most often analysed objects in our laboratory, hosted by the Opificio delle Pietre Dure in Florence, one of the most prestigious restoration centers in Italy. Their nearly flat morphology with very fine features, e.g., paint detachments, requires very high-performance devices to allow for the detection of details at the micrometric scale. Therefore, among the various parameters, we evaluated the instrument axial resolution, which is never provided by the manufacturer but is essential not only for the proper instrument choice but also to qualify the quality/reliability of the 3D model.

Considering that the restoration of an artwork often takes a long time (up to ten years or even more), the instrumentation used to document the repair work is inevitably subject to replacement and/or evolution. Therefore, an additional aim of this work is to provide a measurement protocol for comparing the features essential for a faithful reproduction of the 3D characteristics of an artwork. The authors are not aware of papers reporting and discussing the performance of such a variety of instrumentations, which we were able to acquire thanks to the SHINE project (see Funding Section), aimed at strengthening the Italian E-RIHS infrastructure for cultural heritage. The only comparisons we found in the literature concern photogrammetry, considered the cheapest technique for shape reproduction.

Technological progress will undoubtedly produce devices with an increasingly higher performance; hence, this work shapes up to be a sort of contemporary screenshot of the potentialities of 3D survey in cultural heritage with a set of devices currently on the market, except for the micro-profilometer, whose optical head is definitely out of production and is therefore no longer replicable.

## Figures and Tables

**Table 1:** Technical specifications of the four instruments used in this work (“/” means data not provided by the suppliers’ data sheets).

	EinScan Pro 2x	MICRON3D Color	FARO Focus Premium	ConoPoint-10		
	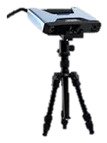	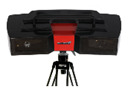	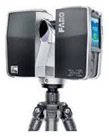	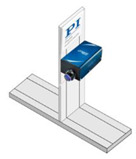		
Focal length	/	/	/	50 mm	100 mm	200 mm
Spot diameter *	/	/	/	37 µm	63 µm	105 µm
Field of view	/	30 cm × 40 cm	300° vertical/ 360° horizontal (0.009° sampling step)	/	/	/
Working distance	510 mm	70 cm	/	44 mm	95 mm	200 mm
Depth of field	200 mm	20 cm	0.5–350 m	8 mm	35 mm	125 mm
Reproducibility (dynamic)	/	/	/	1 µm	4 µm	25 µm
Accuracy	0.04 mm	60 µm	2 mm	6 µm	15 µm	70 µm
Distance between points	0.16 mm	80 µm	1.5 mm (scanning dist. 10 m)	=sampling step		
Point density	/	150 pp/mm^2^	/	/	/	/
Scan efficiency	Better than 0.5 s	/	/	/	/	/

* At beam waist.

**Table 2:** Micro-profilometry acquisition parameters.

Sample	Focal Length (mm)	Sampling Step (µm)	Scanned Area (mm^2^)	Acquisition Time (min)
Acrylic paint mock-up	50	20	53 × 50	75
		50	125 × 260	184
		100	53 × 50	15
	100	50	53 × 50	30
		100		15
		200		7
	200	100	53 × 50	15
Canvases 1 and 3	50	20	55 × 108	120
		50		51
		100		25
	100	50		51
		100		25
		200		12
	200	100		25
Canvas 2	50	20	60 × 60	90
		50		36
		100		18
	100	50		36
		100		18
		200		10
	200	100		18
Plaster statue	100	50	75 × 130	80
		200		20
	200	100		41

**Table 3:** Details of the point clouds obtained with the micro-profilometer, MICRON3D and EinScan structured-light scanners and the FARO Focus phase-modulation scanner.

		Micro-Profilometer				MICRON3D	EinScan	FARO Focus
		f50			f100			
		s20	s50	s100	s200			
Acrylic paint mock-up	Sampled area (mm^2^)	53 × 50						
	N. points	6,593,830	1,208,797	263,375	65,076	341,174	80,174	91,783
	Density (pp/mm^2^)	2539 ± 88	441 ± 14	110 ± 4	25 ± 1	127 ± 8	27 ± 2	25 ± 5
Canvases 1 and 3	Sampled area (mm^2^)	55 × 108						
	N. points	14,848,389	2,376,866	595,395	149,302	771,558	152,992	206,430
	Density (pp/mm^2^)	2368 ± 212	381 ± 34	95 ± 9	24 ± 3	125 ± 11	26 ± 2	27 ± 5
Canvas 2	Sampled area (mm^2^)	60 × 60						
	N. points	9,005,771	1,442,398	361,162	90,592	535,987	101,398	121,415
	Density (pp/mm^2^)	2448 ± 178	375 ± 25	95 ± 7	24 ± 2	145 ± 10	27 ± 1	30 ± 3
Plaster statue	Sampled area (mm^2^)	30 × 30						
	N. points	N.A.	N.A.	N.A.	25,939	187,209	35,199	58,362
	Density (pp/mm^2^)	N.A.	N.A.	N.A.	19 ± 6	134 ± 16	27 ± 6	35 ± 11

**Table 4:** Mean thickness and standard deviation for EinScan, MICRON3D and micro-profilometer equipped with 50 and 100 mm lenses and theoretical values of each painted area. The five areas of increasing thickness are numbered from 1 to 5 and coloured as they appeared on the conditioned surface.

		Thickness (Mean ± St. Dev.) [µm]				
	Pigment’s Region	1	2	3	4	5
Instrument/ Theoretical Value						
MP_f50_s20		47 ± 13	97 ± 18	146 ± 14	217 ± 15	262 ± 18
EinScan		49 ± 28	97 ± 28	147 ± 24	216 ± 24	297 ± 38
MICRON3D		70 ± 31	117 ± 41	168 ± 49	238 ± 42	315 ± 46
MP_f100_s100		57 ± 40	101 ± 40	147 ± 41	213 ± 43	285 ± 47
Theoretical value		50	100	150	200	250

## Data Availability

The data presented in this study are available on request from the corresponding author.
